# Leadership, Social Capital and Coastal Community Resource Governance: the Case of the Destructive Seaweed Harvest in West Bali

**DOI:** 10.1007/s10745-016-9832-y

**Published:** 2016-06-07

**Authors:** Carol Warren

**Affiliations:** Asia Research Centre, Murdoch University, Perth, Australia

**Keywords:** Tragedy of the commons, Leadership, Community-based resource governance, Rules-in-use, Scenarios, Bali

## Abstract

This paper concerns resource governance in a remote Balinese coastal community, which faces severe environmental challenges due to overexploitation and habitat destruction. It explores some of the issues raised in ‘social capital’ debates regarding leadership and public participation toward sustainable natural resource governance. Given the strength of Balinese customary law and the high degree of participation required in the ritual-social domain, Bali represents a model context for examining these issues. Through a case study of destructive resource exploitation and evolving rules-in-use, this paper analyses the ambiguous role of ‘bonding’ social capital and the complexities of negotiating collective action on environmental problems where conflicting interests and dense social ties make local action difficult. The paper finds that a more complex appreciation of vertical (authority) and horizontal (solidarity) relationships between leaders and ordinary villagers is required, and that a more nuanced institutional bricolage and exploratory scenario approach to analysis of evolving rules in use would enhance associated policy interventions.

## Introduction

Assumptions of the social capital thesis, that shared values and dense associational ties contribute to good governance, are currently being imported into natural resource management literature as the practical failures of state and international environmental agency interventions mount (Berkes and Folke 1998; Pretty and Ward 2001; Agrawal 2002; Ostrom 2010; Guitierrez *et al.*^2011^; Mitchell *et al.*^2015^). Given their policy-oriented focus, work in the area of ‘commons’ scholarship has tended to follow Putnam *et al.* (1993) in seeking horizontal ‘bonding’ social capital conditions in communities that could contribute constructively to collective action on natural resource management. These approaches have been subject to criticism for their tendency to ignore the internal structural complexity of communities, in particular the different interests of local actors and especially those elites in a position to capture the benefits of resource access (Agrawal and Gibson 2001; Mosse 2006; Bebbington 2007; Li 2007; Warren and McCarthy 2009; Fine 2010). Following Bourdieu (1986) on the other hand, approaches to the question of ‘elite capture’ tend to assume that those with strategic positions and superior resources – including the extended networks that are associated with ‘social’ capital formation and the ‘symbolic’ capital that can be acquired through various forms of culturally ascribed and achieved status (titles, credentials, official positions) – will use that capital to appropriate disproportionate benefit to themselves (Platteau 2004; Abraham and Platteau 2011). This approach tends to color most discussion of ‘leadership’ in the critical social science literature.

These are particularly acute issues in communities dependent upon marine resources, which are regarded as more or less classic ‘open access’ regimes because of the difficulties of governing the mobility of fish resources and fishing populations (Marschke 2012: 39–56). Responding to the global decline of fish stocks and marine habitats compounded by the effects of climate change, will necessarily be dependent upon developing much more effective governance regimes from global to local scale (Lockwood *et al.*^2012^). At local level, the problems of community participation, leadership and the negotiation of horizontal and vertical structures through conservation interventions becomes an important consideration in establishing the legitimacy and ultimately the long-term effectiveness of sustainable co-management regimes (Bené *et al.*^2009^; Guitierrez *et al.*^2011^).

In Indonesia considerable attention has been given to customary (*adat*) governance mechanisms and resource management practices that suggested possibilities for building networks of locally managed sustainable fisheries (Thorburn 2000; McLeod *et al.*^2009^). These offered the prospect of linking local protected no-take areas and restricted harvesting zones to wider marine protected area networks in which locally managed resource use could be negotiated to take account of customary rights and practices as well as livelihood needs. Critical perspectives on this customary governance approach revolved around the complexity of community structures and failure to recognize how internal inequalities complicate these conservation approaches (Bailey and Zerner 1992; Pannell 1997; Brosius *et al.*^2005^; Afiff and Lowe 2008; Davis and Ruddle 2012).

Elinor Ostrom’s most recent work on commons governance indicates a much more qualified perspective on the institutional determinacy of vicious and virtuous cycles in the relationship between structure, agency and institutional design (Ostrom 2010: 163). This paper explores the importance and limitations of the narrow institutional focus that has been characteristic of community resource management theory and policy to date, and consequently pursues prospects for the broader, more flexible and context sensitive approach suggested by Frances Cleaver’s (2002, 2012) ‘institutional bricolage’ framework.

The Bali case study elaborated below serves to qualify a number of assumptions associated with the social capital thesis and allied conservation policy approaches. First of all, the case shows that high levels (especially of the ‘bonding’^1^ type) of social capital may actually inhibit the resolution of commons governance issues through collective action under certain conditions. In the Bali case leadership was constrained by horizontal social ties, although a high level of ambivalence among leaders as well as ordinary villagers is evident because of the pervasive conflict between short-term economic needs and long-term environmental wellbeing. It is argued that an institutional bricolage framework offers a realistic approach to the equivocation arising from this ambivalence. In this more context specific analysis and policy framework, leadership – exercised by elites as well as ordinary villagers, insiders as well as outsiders, in formal as well as informal contexts – engages vertical and horizontal agencies in a more polycentric, open-ended and ongoing negotiation process toward improved resource governance.

## Bali Coastal Community Case Study

Given the strength of Balinese customary law (*adat*), and the high degree of participation traditionally required in the ritual-social domain, Bali represents an ideal-typic case for examining questions central to the adaptation and extension of customary governance practices to contemporary community-based resource management (Warren 1993, 2005). This case study examines limits to the extent to which the horizontal dimensions of customary governance regimes may be transferred to contemporary natural resource management and challenges simplistic assumptions regarding the role of leadership and elite control of material and institutional resources (Knudsen 2013). The paper argues that the complexity of vertical and horizontal relationships in the Balinese context points to the importance of considering the interdependence between both dimensions of this binary in the literature on social capital described above and elaborated in the introductory chapter to this volume (Warren and Visser 2016).

The research for this case study formed part of a wider comparative project on ‘Social Capital, Natural Resources and Local Governance in Indonesia’.^2^ It involved mixed-method quantitative (survey) and qualitative (participant observation and interview) approaches. A random sample household survey, including questions on socio-economic status, involvement in village level decision-making, and attitudes to local environmental issues was conducted with 40 households, geographically distributed across the 5 hamlets in this village. Field research took place over annual periods of several weeks to a month from 2010 through 2015. Recorded in-depth interviews with local leaders, including village and hamlet heads, customary community leaders, government agents and fishers’ cooperative members provide the source of direct quotations translated by the author, and give voice to the range of perspectives on resource decline in this village. An ethnographic approach to researching local resource issues enabled analysis to take account of the specificities of the local needs, understandings and practices that in this case led to the destructive seaweed harvest of 2012, the long period of indecisive debate concerning its regulation, and the complex institutional ‘bricolage’ scenario which appears to have brought resolution.

The location of the case study is the village of Perancak, a remote fishing community on the far west coast of Bali, until recently classified by the government as a ‘*desa tertinggal*’ (socio-economically ‘left-behind village’). Located along a narrow strip of coast between the Perancak River and the Bali Strait, a three-hour drive from the provincial capital of Denpasar, this village faces a number of extremely serious and interacting conservation issues affecting its primary coastal resource base. These include overfishing, coastal erosion, mangrove destruction and the endangered species trade. Over the last two decades there have been responses of varying effectiveness to some of these complex issues of environmental degradation through a combination of local customary (*adat*) and administrative government regulation, involving interventions by external government agencies or non-government organizations. Among examples of collaborative engagements between the local community and external stakeholders towards improved resource governance have been efforts to deal with mangrove destruction and sea turtle conservation.

Mangrove destruction for shrimp pond development and firewood collection is generally regarded now to be effectively controlled by a combination of customary village regulations (*adat*) and the intervention of a joint government and internationally funded fisheries research center, SEACORM,^3^ located near the village. Although there is still some low-level firewood collection, local customary regulations and external agency involvement are regarded as having successfully halted mangrove clearing – although not before a large area had been converted to shrimp ponds.

The second instance of partially successful co-management has been the establishment of a sea turtle conservation group in Perancak village. Bali has been the center of a now illegal (but still substantial) sea turtle trade. A WWF campaign since the 1980s brought about significant pressure for state legal action to protect the tourism industry from threatened international boycotts. At the instigation of a Perancak family group that had traditionally hunted turtles, a WWF-supported turtle nest protection program was established in the village in the 1990s. Although the project faces problems of funding continuity and compliance with best practice guidelines (Ross 1999), it has nevertheless survived for more than a decade and is to date the most successful nest protection program on the island.

Other significant environmental issues facing this community that have had neither effective local action nor serious attention by any level of government or outside conservation body to date, include coastal erosion, unregulated tourism development and overfishing.

GPS data shows that 60 ha of Perancak coastline has been lost between 2000 and 2014 (pers com., Made Puriati, 16.7.15). This meant that a number of households were forced to find residential land elsewhere in the village and that small artisanal fishers’ outrigger mooring sites are threatened. This ongoing loss of coastal land has spurred another socio-economic problem, driving the sell off of land along the coast to investors for tourism and real estate development. The transfer of village coastal land ownership to foreign interests also poses problems of beach access and landing space for small fishers’ boats. While some concern with threats to cultural and environmental values in the community has also arisen, the potential for low-skilled youth employment in villas along the coast is seen as an important alternative prospect given the declining fishing industry.

Most serious for the long-term survival of the local fishery and fishers’ livelihoods, are the unsustainable fishing practices associated with the large commercial purse seine fleet that operates out of the Perancak River. These boats, which have been based in Perancak since the 1970s, serve several factories on the other side of the river, processing a range of fish products from animal feed to canned sardines for the international market. The purse seine boats take huge volumes of juvenile fish in illegal 1 cm mesh-size nets.^4^ They frequently operate illegally in the inshore zone, where they compete with small-scale artisanal fishers using traditional outriggers (*jukung*). Although mostly owned by non-Balinese from neighboring villages, and despite longstanding tensions over inshore fishing and net sizes, the fact that large numbers of Balinese villagers (including many small *jukung* fishers) are employed as part-time crew on the purse seine boats, means that short-term conflicting interests inhibit what local action might otherwise pressure for law enforcement.

During the 2010–2011 season there was still some debate in the village over the extent to which these practices accounted for the severe drop in the sardine catch, from which the commercial fishery has not recovered to date.^5^ Past protests by local small fishers’ cooperatives and calls on the government to enforce existing regulations on gear and catch zones, and to implement the district government agreement to limit the size of the purse seine fishing fleet through licensing, had until 2014^6^ fallen on deaf ears. Recorded landings of fish at the nearby official auction centre (TPI), where the commercial purse seine fleet land their fish, had risen from 6.6 million kg in 2006 to 21.8 million kg in 2009, but dropped dramatically to 11.6 million kg in 2010 and 6.0 million in 2011, when sardines, the mainstay of the Bali Strait fishery, comprised less than a quarter of the catch.^7^

## Local Governance: Customary and State

Customary law (*adat*) is one local governance mechanism in Bali that is regarded as a potentially effective approach for dealing with environmental issues, especially in the post-authoritarian period of decentralized governance in Indonesia. *Adat* can be a powerful tool whenever it is possible to conjure consensus and impose customary sanctions that for Balinese have material, social and spiritual implications. But the powers of local customary regulations do not extend beyond the Balinese community that makes and enforces them. And so, some issues, such as regulation of the large purse seine fishing fleet and of tourism development, are largely beyond direct local control. Where local management is possible, the difficulties of achieving the community consensus that is prerequisite to effective imposition of rules and sanctions cannot be underestimated either, even in this high social capital context.

Revision of village customary laws (*awig-awig*), carried out periodically through a process of lengthy community deliberation and eventually consensual determination, theoretically offered the opportunity to tackle some of these issues. After a controversial process of revision, that did not include the extensive public consultations normally required, the provisional new village customary regulations (*awig-awig pasuara*) drafted in 2011 barely touched on most of the serious resource governance issues described above. The process and outcome of the *awig-awig* revision exercise give some indication of the kind of internal tensions that critics of commons literature point to regarding the need to revise ‘romantic’ constructions of the ‘community’ concept and to recognize competing interests and the potential for elite capture of local resources, including customary governance itself (McCay 2001; Brosius *et al.*^2005^; Davidson and Henley 2007; Thorburn 2000).

Beyond the inclusion of earlier regulations on mangrove exploitation, the most recent revisions to the customary regulations for Perancak either skirt controversial natural resource management issues or uncharacteristically refer their enforcement to state law. The failure to address these issues and the lack of customary due process was regarded by some villagers as indicating ‘elite capture’ of the customary decision-making process. The recently elected *adat* head of the village (*Bendesa*) was a relative of the then official village head (*Perbekel*) in the dual customary (*adat*)–state (*dinas*) local governance structure that has prevailed in Bali since the colonial period. Both *adat* and *dinas* village heads were among the few Balinese that owned purse seine boats, until the crash in the sardine fishery in 2011, when the *adat* leader could not pay his debts and had to sell off his boats. Both official and customary village heads were democratically elected, although both were believed to have ignored serious issues that conflicted with their private interests. An example of outright elite capture of development resources was the use of government community development grants (PNPM) to purchase four flat bottomed sampan owned by the administrative village head for use as tourist boats. More appropriate would have been an arrangement to use the traditional *jukung* outriggers, which would showcase local heritage while providing income to small-scale fishers. But with this exception, most villagers interviewed regarded the distribution of development funding for projects and employment through the core government community development program (PNPM) as fair, although many disliked the administrative village head’s arrogant manner, which eventually led to defeat in his bid for re-election.

Beyond the vested interests of elected village officials, we need to consider also the complexity of the interconnections, dependencies and conflicting interests that we have come to recognize as characteristic of all ‘communities’ if we want to understand why collective action on critical environmental issues does not necessarily arise despite these being serious local concerns. Under normal circumstances cohesive communities often tolerate rule bending and permit everyday ‘business as usual’ practices to proceed in the interests of stable social relations – although not without the gossip, suspicion, righteous indignation and other forms of contestation born of competing perspectives and interests that pervade even the most ‘homogeneous’ and ‘bonded’ of *adat* villages. Local leaders and powerful elites^8^ cannot be completely insensitive to these horizontal rumblings that erupt in periodic outbursts at village meetings, in jokes and tirades in the local coffee shop, or in satire that has the quintessential appeal of a strong subversive comic tradition in Balinese artistic performance, a requisite part of all major ceremonies in the annual ritual cycle.

Exploring this theme further, the paper now turns to an example of collective action failure on a significant environmental issue in this high social capital context. The incident described below raises important questions about the relationship between horizontal and vertical dimensions of local social structure that are of wider significance than much of the literature on either ‘elite capture’ or exemplary leadership (Guitierrez *et al.*^2011^; Knudsen 2013) in cohesive contexts suggests.

## Collective Action Failure: Ravaging Coastal Seaweed Beds

In 2012, the year following the collapse of the Bali Strait sardine fishery, a trader came to Perancak from Surabaya offering villagers cash^9^ to harvest the natural seaweed bed along the eastern end of the Perancak coastal strip. Seaweed (known locally as *bulung jaje*) is used for making agar-agar, cosmetics and other products, and had a high market price at the time. Because of the drastic decline in the local fishery during the previous two years, villagers were desperate for income, and joined in an open harvest, ravaging the previously undamaged area of the village coastline of its seaweed beds. Two months later, in May 2012, rough seas and unusually high waves ripped several metres from the already seriously eroding coastline in precisely the area where the seaweed harvest had taken place. Discussions with local fishers, fishing cooperative and hamlet leaders reveal the complexity of the ‘drama of the commons’ that ensued.

NK, a fishing cooperative member and head of the administrative hamlet (*banjar*) that stretches along the affected strip of coastline found it impossible to intervene directly in the face of conflicting economic needs and cultural values. He believed there was need for both formal state and customary regulation:

The traders came with trucks from Surabaya offering cash to collect the seaweed (*bulung jaje*). People used to collect the *bulung jaje* flower for cooking before, but there was never anyone to sell it to. People from all over the village joined the harvest. In two weeks it was finished. There is only stubble left. I didn’t dare say anything. I couldn’t prohibit it. There has been a long period of scarcity this fishing season and everyone would protest. I certainly couldn’t do that in the atmosphere of a forthcoming election. People came from SEACORM to watch but didn’t do anything either. There are no regulations prohibiting this, not like there are for coral or mangrove. Only later did the community regret it…. In fact, even while it was going on some of those who had participated in the harvest, came to me telling of dreams they had. One of them told me that he had been visited by a tall, old man in his dream. The spirit said: “Why are you bothering my place”, and he stopped [harvesting seaweed]. Three villagers came to me with similar stories of warnings they had in their dreams. But I couldn’t really do anything. Only now are the community feeling the effects. The stubble is still there, so maybe it will grow back. The fish used those areas for hiding from prey and laying eggs. The sea turtles used it as food, and the roots hold the sand in place. … Coastal abrasion now is so bad everywhere, the whole of Bali is going to sink and be destroyed, not just Perancak … if the government doesn’t quickly do something about protecting coastal areas. The problem is there is no regulation on seaweed collection, only on coral, the use of bombs, and mangroves. There needs to be both *adat* (customary) and *dinas* (state) regulation to be strong enough.

(Interview with NK 22/07/12)

A former customary (*adat*) hamlet leader and founder of the turtle conservation project believed that there was collective moral responsibility to take action, irrespective of the lack of formal regulations. He had been quietly promoting the idea of a small no take zone in the area of the seaweed beds where an underground cave, rock and coral reef formation remained relatively intact, although he no longer had a formal position of authority from which to do more than privately criticize his fellow villagers. He described what happened with disdain:

…Without the sea grasses the sun’s rays go straight to the coral and rocks, and fish won’t be able to lay eggs there. The villagers had the excuse that their stomachs were hungry. I said, “You can’t take rocks [coral] with that excuse. Aren’t we stirring up satan? If we used that excuse to take away the coral, the world would go to pot … We can’t go around killing people with the excuse that we are hungry.” I don’t know why [we] Bali Hindus are always talking about the Tri Hita Karana principle of balance in the relationship between the gods and nature and other people, but we never actually practice it. No need for God to annihilate the earth, what a pity! – no need! Let mankind do that to themselves if they abuse [nature]. (Interview with WT 25/07/12)

The head of one of the small fishers’ cooperatives in the village best expressed the situational ambivalence that is an under-recognized aspect of commons dilemmas, and that undoubtedly explains a good part of the uncertainty that characterizes so many outcomes in contested situations:

The impact was severe. Fish need the seaweed beds to lay eggs, turtles for food. Before the seaweed beds were finished off, they would moderate the coastal current. So of course the erosion now is more extreme. Uhh – tsk! [Shook his head] But if we fault our fellow fishers, they would be insulted and angry. Actually this is the responsibility of the Department of Fisheries: if they had come down, then we could have done something about it. … Among one another, it would be explosive to go around giving warnings. From the village head there was only talk about where else to look [for seaweed]. SEACORM said that if the seaweed beds were finished off, there would be an effect on the water movements and the fish wouldn’t be there. But the fishers didn’t pay any heed. They needed money. How many tons were taken away? Maybe 50 tons. The blades were as long as my arm. Perhaps I would have to be reincarnated four times to the earth before they could grow back to their original length. But rather than sit dumbfounded, I joined in the harvesting. So I saw it all. It wasn’t that we didn’t all know the effects of it; we felt forced to because of the situation. … There was no money.

(Interview with KS 21/7/12)

These were all respected local leaders, themselves fishermen – thoughtful, world-wise, straight talking, primary and lower secondary school educated – who generally fit the Balinese ideal model of local leadership. In this instance they present themselves as ‘captured’ by horizontal bonds as the dire economic situation pressed for priority over environmental concerns. They saw their position as characteristic of model egalitarian leadership in customary institutions, likened in a Balinese proverb to ‘leading a flock of drakes – you get all flack and no eggs!’ In their leadership roles as first among equals, they felt unable to act to restrain the free for all exploitation of marine resources (cf Li 2014: 12).

The depth of competing subject positions (in particular of short- versus long-term, and individual versus collective interests) for those caught in such dramas of the commons is expressed by the last quoted fishing cooperative leader, who admitted joining the harvest himself against his better judgment. Despite his own participation, he was a supporter of the proposal for a no take zone in the area of the harvest and of better government enforcement of existing regulations, demonstrating that leaders’ individual ‘interests’ are not necessarily more coherent and consistent than those of the communities they represent. His account starkly reveals Hardin’s classic ‘tragedy of the commons’ scenario where individual short-term rational action conflicts with the long-term collective action required for protecting environmental resources in the common interest. All agreed that the critical issue was the difficulty of achieving an active consensus on establishing restrictive rules in the face of short-term need. Even the District Fisheries Department official (Interview WK 25/07/12) referred to both horizontal sensibilities and vertical authorities as his excuse for not intervening. He said he did not want to prevent the seaweed harvesting out of human sympathy for those facing economic difficulty. And because it was neither technically illegal nor on the radar of the ‘higher ups’ in the bureaucracy, he had no real power to do anything, although he also observed that the long-term impacts would certainly be damaging.

These responses underline the importance of taking seriously the ever-present (but usually short-term) conflict between livelihood and sustainability discourses, and the multiple identities and positioning of actors, which post-structural perspectives have sensitized us to acknowledge, but which tend to slip from view in legal and policy debates. The hybrid identities and competing interests from which actors negotiate their positions on natural resource management issues appear to be critical to understanding the ambivalent and equivocal tendencies toward collective action/inaction that contribute toward or undermine the potential for constructive solutions to environmental problems – in this case an acknowledged failure to deal with already well recognized local environmental stresses in a crisis driven context. In our 2011 research project survey of a geographic cross-section of 40 households in the village, undertaken the year before the seaweed harvest event, 29 of 40 (73 %) respondents nominated coastal erosion as a serious environmental issue that needed to be addressed (2011 Survey: Q500). In response to a general question on the most important challenges facing the village, environmental issues ranked highest (14 of 28 responses), above combined educational (2), economic (6), infrastructure (1), political (4), and other (1) issues nominated by respondents (2011 Survey: Q304). Yet this relatively high level of awareness did not immediately translate to a precautionary response to the seaweed harvest opportunity in 2012.

Power lies within Balinese communities to alter some aspects of the environmental challenges that face them through both customary and official institutional means. The case of the seaweed harvest suggests that the ‘tragedy of the commons’ is in many contexts as much an effect of horizontal relationships among fellow villagers as of vertical relations between state or customary authorities and ordinary community members. Responses from the range of local leaders quoted above indicate that scholarly concern with ‘elite capture’ must also take account of ‘captured’ leadership who face serious political risks and personal pressures where different needs and interests threaten solidarities. Strong horizontal bonds in situations of potential conflict may confound efforts to challenge, recruit or constrain fellow subalterns to effect what Hardin (1968: 1247) described as “mutual coercion, mutually agreed” – his one local egalitarian-democratic option for commons governance beyond the state and private models that have dominated policy approaches until recently. It is argued here that the relatively high level of bonding (internal solidarity) social capital (Woolcock 2001), assumed to provide the very mechanisms that might construct a virtuous cycle for engaging collective action, at the same time stood in the way of addressing some of the key environmental issues in this village. Given the high level of ambivalence and leadership impotence reflected in these accounts of the seaweed harvest, what prospect for resolving emergent environmental issues requiring legitimate authority and mutually agreed rules?

## Conservation Scenarios

In one of her last articles on institutions for collective action, Nobel Prize winning economist Elinor Ostrom (2014), whose work has focused so prominently on common resource policy issues, calls for research into the processes of ‘rule evolution’. There is considerable reason to question the narrow and reified focus on institutional rules that pervades so much of the literature on environmental governance (Cox *et al.*^2010^). Yet the timely emergence and deployment of ‘rules-in-use’, as Ostrom (2005, 2014) refers to this key component of commons institution building, were regarded by local leaders as completely lacking in the seaweed harvesting ‘tragedy’ in Perancak.

Ostrom (2010, 2014) struggles in this most recent work to come to terms with the apparent indeterminacy presented by the mixed body of evidence on local resource management issues. She calls for detailed research that tracks the processes of decision-making and the evolution of rules-in-use over time. To this end she proposes that we use this research to model a range of community resource governance scenarios derived from empirical cases to explore where and how tipping points in one direction or another occur. Mitchell *et al.* (2015) argue that scenario planning can also be a valuable tool in the repertoire of approaches that enable adaptive co-management regimes to turn vicious into virtuous circles by bringing together scientific and experiential evidence, local social values and cultural sensibilities into formal institutional frameworks for environmental protection at all levels of governance.

Perancak presents a small but interesting case study for the kind of scenario exploration that Ostrom (2010: 164) suggests offers a way forward. Combined with perspectives from political economy and critical discourse analysis in such cases, and sensitized by the bricolage perspective proposed by Cleaver (2012), this approach requires us to track over time the range of perspectives and collective action responses among local actors and across institutional frameworks affected by the multiple serious environmental threats faced by this community. It requires us to trace the points at which conflicting interests, ambivalent positions and alternative options lead local actors to engage or disengage, accommodate or resist, become active citizens or desert the field.

Rather than solely focusing on the more or less formal processes supposed to produce clearly legible outcomes, Cleaver’s (2002, 2012) elaboration of institutional bricolage as a complementary perspective to the rather more narrowly instrumental and functional tendencies of the institutionalist paradigm, recognizes less conscious and more conflicted processes, which enable us to understand why purely ‘rational’ simplifications fail (2000; 2002: 15–16). It is also an approach that recognizes the importance of the time factor, of tenacity, commitment and responsiveness to the unexpected that have become critical themes in recent common pool resource studies. In the ongoing saga of Perancak’s coastal degradation, we need then to seek out those who have or could play roles as institutional ‘bricoleurs’ (Cleaver 2002, 2012), through formal or informal interventions, to contribute to effective and culturally relevant articulation of evolving rules-in-use.

In the case of the ravaged seaweed beds, the few villagers who did not join the seaweed harvesting frenzy associated that site with sacred/dangerous forces, apparently connected with its special ecological features. Their interventions did not immediately affect the destructive harvest, but did apparently touch on sensitive cultural dispositions. According to the former customary community leader, WT, the reason this area along the Perancak coastal strip had remained in relatively pristine condition had to do with local cultural sensibilities associating supernatural forces with the rock-cave-coral formation that is fringed by the seaweed beds. The special character of the site was widely remarked upon, and the area was respectfully avoided by most local fishers.

Following Ostrom’s suggestion that we give close attention to the development of responses to specific cases of commons management, this paper now considers a spectrum of scenarios to model the range of potential pathways to improved governance of coastal resources for Perancak. These involve actors and institutions already in play, but place different emphases on the articulation of vertical and horizontal, formal and informal governance relations, and implicate different time frames and institutional resources.

*Scenario 1 State government alliance with international agencies* (vertical, top-down, formal management approach) – The global agency Conservation International (CI) is in the process of promoting establishment of an extensive Marine Protected Area (MPA) network to encircle Bali, mainly through lobbying provincial and district government departments to enact official MPA declarations. This has been given traction by the national plan, also pushed by the three collaborating international conservation agencies (Conservation International, World Wildlife Fund, The Nature Conservancy) under the Coral Triangle Initiative, to expand Indonesia’s network of MPAs to 20 million hectares by 2020.^10^ Past experience, however, demonstrates that MPA declarations are unlikely to have meaningful outcomes for coastal conservation and marine biodiversity in the absence of local engagement and support for enforcement (McClanahan *et al.*^2006^). Conversely, local efforts will be difficult to make effective without at least official recognition, and ideally an active commitment to shared governance that has to date been lacking (Persoon *et al.*^2003^; Lockwood *et al.*^2012^). Undoubtedly, in the case of the Bali Strait fishery, state enforcement of existing regulations on zones and fishing gears would be a significant starting point for building the trust in formal governance institutions, necessary also as a building block for shared governance agendas.

*Scenario 2 Local customary regulation with local NGO support* (bottom up, culturally framed, community-based management approach) – Local knowledge and the folk sensibilities to the site of spiritual significance that troubled the former customary leader (WT) and several villagers who resisted the seaweed harvest, could be linked with the efforts of a local NGO that has a community-centred approach to popularize this site as a locally managed no-take area and re-enforce knowledge and awareness of connections between religious, cultural and biological diversity values. Some steps in this direction took place when a local NGO that had previously been involved in community mapping at Perancak brought the director of the Locally Managed Marine Area (LMMA) network to the village to discuss the former *adat* leader’s suggestion (WT quoted above) of establishing a no-take area around the spiritually significant site.^11^ That promising engagement did not have any immediate result because LMMA hesitated to stretch its scarce resources in the absence of strong local consensus or evidence of customary institutional support. Although use of *adat* regulations had contributed to stabilizing mangrove exploitation, *adat* institutions were unlikely to deal with the seaweed harvesting or unregulated fishing in the short term because of conflicting interests and the tight grip that the related customary and administrative village heads held at that time over village government.^12^ Nonetheless, more concerted attention to the informal aspects of local environmental perceptions through this approach might have bridged some of the tensions that emerged in the months following the harvest, and cultural sensitivities undoubtedly did feed into the subsequent turn of events that eventually halted the harvesting practice three years later.

*Scenario 3 Institutional bricolage* (combining the potential of vertical and horizontal institutional strategies represented in scenarios 1 and 2, but giving more attention to process, meaning-making and actors’ ambivalent subject positions in complex structural contexts) – Building upon the formal institutional components represented in Scenarios 1 and 2, while recognizing their limitations, becomes the starting point for a more open-ended trial and error approach to changing local practices. Cleaver’s (2002, 2012) practice theory conceptualization of institutional construction through bricolage – is focused more on the *ad hoc* and relatively less predictable confluence of processes and events that influence agency and shift or confirm existing practices and institutional arrangements. A bricolage approach seeking to bring into conjuncture some combination of existing institutions and agencies, would explore a diverse range of potential accommodations across governance scales. The hybridity of actors’ identities and interests brings into play economic needs and calculating interests, as well as cultural associations, folk sensibilities, and social relational desires for respect and regard, which can be used to re-enforce or revise governance practices.

The aim of leadership in an institutional bricolage scenario is to facilitate convergence through the combination of formal and informal processes that Cleaver’s work is at pains to expose: “The evolution of collective decision-making institutions may not be the process of conscious selection of mechanisms fit for the collective action task (as in Ostrom’s model), but rather a messier process of piecing together shaped by individuals acting within the bounds of circumstantial constraint” (2002: 17). Cleaver’s institutional bricolage embeds arrangements in “….networks of social relations, norms and practices … in which maintaining social consensus and solidarity may be equally as important as optimum resource management outcomes” (2002: 17). These networks and norms include the very horizontal social relations of bonding social capital that inhibited collective action against the damaging natural seaweed harvest, but could equally underpin solidary collective action responses under more accommodating circumstances (Warren 1993). At the very least, they prompt consideration of the reciprocities and equities that must be addressed to enable more positive and enduring outcomes.

The bricolage scenario is undoubtedly prone to the same unintended consequences and perverse outcomes that plague planned formal institution-building approaches summarized in Scenarios 1 and 2. But if pursued seriously, it opens conservation and development engagements to a more responsive repertoire of options and broadened concept of leadership as changing circumstances present themselves. In the case of particularly activist or charismatic leadership, decisive outcomes might be conjured, but in the everyday world of local governance progress mostly presents itself as incremental ‘improvisations’ pieced together “to address everyday challenges” (Cleaver 2012: 46). This approach would not rely on a one-size-fits-all template simplistically represented here by Scenario 1 or 2, but an actor and process-focused conjunctural approach that recognizes competing structures and forces, and would ideally be paralleled by a more critical and flexible action research agenda. The kind of social and cultural embedding that Cleaver speaks of could also make the underpinnings of emerging responses more durable and adaptable in the long run. The bricolage approach recognizes that what does or does not work at one place and time may yet find a place in different temporal and spatial conjunctures. This approach fits well with the growing interest in adaptive co-management and social learning processes, but requires a more organic approach to the informal aspects of these processes and presupposes less bounded time frames.

## Reflections on ‘Institutional Bricolage’ and Emerging ‘Rules-in-Use’

Something approximating a modified version of the composite third scenario seems to have emerged in the wake of an abrupt turn in the official position on fisheries policy as a result of the 2014 Indonesian national election. Under the populist President Widodo (who was elected from outside the national political establishment), food security, sustainability and the situation of small-scale producers have been highlighted as new policy priorities, replacing the previous government’s narrow focus on productivity increases. The newly appointed Minister of Fisheries, Susi Pudjiastuti, issued regulations banning trawl and purse seine technologies because of their environmental impacts and declared a moratorium on new commercial fishing permits.^13^ She also began hard line law enforcement against foreign vessels fishing illegally in Indonesian waters, a popular intervention with domestic fishers (Jakarta Post 2015a, 2015b).

In the wake of decisive and controversial policies from the new Fisheries Minister, the unrestrained and opportunistic harvesting of the natural seaweed beds that began in Perancak in 2012 halted suddenly in mid-2015. While it may appear that the end to the harvesting of seaweed follows the logic of Scenario 1, with heavy-handed action by formal government authorities determining the outcome, this is not the local interpretation. The local leaders interviewed previously regarded this recent about face as a meeting up of the hitherto unsuccessful efforts of some villagers to restrict or prohibit the harvest with new state policies that were broadly committed to natural resource conservation.

Minister Pudjiastuti’s reputation as a strong-willed, no-nonsense authority figure, under a government that claims to be committed to fair resource access, undoubtedly gave disproportionate symbolic weight to the installation of a sign on Perancak beach spelling out heavy penalties for infringement of the Conservation Act – a maximum of 100 million rupiah (US$10,000) in fines and 5 years jail to any person “taking, felling, possessing, destroying protected plants or animals” (Law#5/1990: §21[1]). Yet the Natural Resource and Ecosystem Conservation Act quoted on the sign has been national law since 1990, and does not include any reference to seaweed as a protected species. In other words, no official change in formal state regulations affecting the harvest had taken place; nor was there any immediate threat to apply this law directly to seaweed harvesting locally. Yet the national context appears to have been a tie-breaker, stimulating conjuncture between local and national environmental sensibilities.

The failure of scenario 1 resulted from a lack of engagement and interest from state authorities and their failure to adapt and enforce even those regulations that were already on the books with respect to fishing gears and zoning – no less the capacity of these distant state authorities or global NGOs to respond to a suddenly arising local coastal management challenge. The failure of scenario 2 resulted from horizontal differences of need, interest and interpretation in the local sphere. Under economic pressure arising from the collapse of the sardine fishery villagers needed this new source of income. Some rationalized that coastal abrasion was an ongoing environmental problem with external causes beyond local control. No consensus was reached and no local authorities were prepared to intervene on the grounds of economic need, social pressures, and equivocal interpretations of long-term impacts.

It remains to be seen to what extent the emerging bricolage scenario will consolidate around better resource governance and sustainable local practice more generally. Uncertainty remains whether enforcement of marine conservation law will be even-handed, in particular whether new regulations prohibiting purse seine fishing will actually be enforced and with what social, economic and environmental consequences. So far changes to the gear and practices of the purse seine fleet appear to have been deferred to future license renewal, and in this regard questions must be raised about the political clout of the owners of the commercial fleet and the political economy of law enforcement in the resource management sphere.^14^

An important lesson that should be drawn from the context rich bricolage approach is that it would be a mistake to regard the seaweed harvest issue as successfully resolved in the absence of efforts to deal with the wider socio-economic conditions that drove villagers to take advantage of this opportunity to improve their livelihoods in the first place. Institutionalization of new rules-in-use for protection of the natural seaweed bed and the legitimacy of the so far implicit ban on harvesting will also depend upon the extent to which the poorest who rely on the small supplementary incomes from seaweed collecting are effectively drawn into local social and economic development programs to support diversification of livelihoods.

Although ‘resolution’ of environmental concerns may appear to have been a largely chance conjuncture of forces in this particular case, it is possible to draw from the case study some suggestion of strategies that could increase the chances of positive conjuncture for the long-term. The PNPM community development program^15^ engaged external facilitators, mainly from NGOs, who worked with an elected village level (male-female) pair of coordinators to assist their communities with development planning. This worked well in some places and not others depending upon the training, experience and commitment of these internal and external roles and their links to local community organizations. In Perancak, for the few years the PNPM program operated, both village coordinators and external facilitators were well regarded. It would be worth experimenting with similarly systematic training and subsidy of a group of civil society actors whose roles would be to stimulate community development and conservation planning, through greater participation of fishers’ cooperatives and other local associations, as well as collaboration and knowledge sharing with other villages facing analogous issues. This could take place alongside action research approaches that introduce participatory exploration of scenario options as a means of seeking out the points at which institutional bricolage might be effected (Mitchell *et al.*^2015^). Whether engineered through external interventions, devised in response to crisis, or conjured from more inchoate sensibilities, fine grained, long-term investigation of these processes would offer important contributions toward a deeper understanding of processes that build constituencies for conservation and sustainable use.

Co-management approaches also need to recognize the importance of going beyond formal institutional frameworks to deal with the complexities of ordinary communities facing sustainability challenges to their livelihoods. In this regard there is all the more potential from seeking out Cleaver’s ‘bricoleurs’ through a more context sensitive approach to the relationship between horizontal and vertical power relations in local communities and by promoting expanded concepts of leadership as well as new spaces for fostering it. Need this ‘institutional bricolage’ rely only on formally recognized ‘leaders’ and ‘elites’? Or might the evolving new institutional brickwork also come from deeper sensibilities and more informal intimations of concern, such as the dream warnings of several Perancak villagers in the seaweed case? The ambivalence of local leaders and the deep-rooted cultural ‘conscience’ of the resistant villagers suggest points of potential convergence between cultural and ecological sensibilities that could contribute toward new ‘rules in use’ (see Cleaver 2000; Warren 2012) in the Perancak case. Driven by desire for social regard or vested interest, by ancestral intimations or other forms of moral suasion to contribute to collective action by tapping into complementary inclinations among their fellows, multiple bricoleurs’ actions could well contribute to convergent sensibilities upon which more stable institutional adaptations could be negotiated. The policy imperatives arising from this approach demand that we broaden analytic frameworks of time and agency. At the very least, attention to conflicting interests and ambivalent identities must be part of this mix of policy, planning and adaptive practice.

## Footnotes

1 Several types of social capital, which refers to high levels of civic engagement posited to contribute to more effective governance, have been identified in the literature sparked by this debate. The 'bonding' type refers to internal solidarity within common interest communities or groups; bridging and linking social capital refer to relationships between horizontally and vertically related groups or institutions respectively (See Warren and Visser 2016 this volume; Fine 2010; Pretty et al. 2003; Woolcock 2001).

2 The research was funded by the Australian Research Council (DP0880961).

3 SEACORM, Southeast Asia Center for Ocean Research and Monitoring. The main objective of this government research station has been the provision of satellite information to facilitate intensive commercial fishing in the surrounding seas. More recently this brief has expanded to include conservation issues.

4 National regulations required the purse seine net mesh be no smaller than 1 inch (Peraturam Menteri Kelautan dan Perikanan No Per. 02/MEN/2011).

5 On the decline of fish stocks in the Bali Strait Fishery, see ACIAR (2011).

6 Potentially significant changes in central government policy that have been introduced since the 2014 national election are discussed below.

7 Data Produksi/Pendaratan Ikan TPI Pengambengan Tahun 2003 - 2009, 2010, 2011, 2014.

8 ‘Leaders’ and ‘elites’ are treated in this paper as partly overlapping categories: the former exercise influence and authority that may be formal or informal, but that are based primarily on socially recognized personal qualities that are ideally disinterested and oriented toward the common good, while the latter are able to exercise authority and/or influence based on their structural political, economic or social status positions that may be independent of personal leadership qualities.(Cf. Amsden et al. 2012)

9 Rp 1000/kg wet and Rp 1,300 (US$ .10-.13) semi-dried seaweed. (interview KS 21/7/12).

10 See http://kkji.kp3k.kkp.go.id/index.php/en/informasi-konservasi/75-targetkonservasi (2012) accessed 05/06/14.

11 See the Steenbergen essay (2016) in this Themed Section of Human Ecology for background on LMMA.

12 That situation changed when the official village head was thrown out of office in the 2013 village elections. But his replacement has as yet shown no interest in environmental management issues. The adat head, also the butt of considerable local disaffection because of his non-consultative style, has meanwhile turned his economic interests to the fish trading business run by his son, which in 2013–14 started to include seaweed trading.

13 See Indonesian Minister of Fisheries and Marine Affairs Regulations 56/2014 and 2/2015.

14 At a national level the tension between conservation and development policies is evident in the blueprint for fastracking development in all major sectors (MP3EI 2011).

15 See McCarthy et al. (2014) for detailed discussion of the PNPM program, which is also discussed in the Lucas (2016) case study in this volume.
